# Midazolam as premedication for less invasive surfactant administration: a prospective single-centre audit

**DOI:** 10.1136/bmjpo-2025-004219

**Published:** 2026-04-01

**Authors:** Liam Willgress, Dina Sava, Fatma Selim, Zina Wells, Paul Clarke

**Affiliations:** 1 Neonatal Intensive Care Unit, Norfolk and Norwich University Hospitals NHS Foundation Trust, Norwich, UK; 2 Norwich Medical School, University of East Anglia, Norwich, UK

**Keywords:** Neonatology

## Abstract

**Background:**

Less invasive surfactant administration (LISA) is now a routinely practised method of administration of surfactant to neonates with respiratory distress. Many centres routinely use analgesia and/or sedation premedication prior to the LISA procedure; however, many different drugs are presently used, and the optimal premedication drug is unknown. We aimed to assess the efficacy and short-term safety of routine midazolam 50–100 µg/kg use as premedication for LISA.

**Methods:**

This was a prospective single-centre audit of routine midazolam use as premedication before the LISA procedure over a 5.4-year period in a tertiary-level UK neonatal unit. We reviewed rates of procedural success, physiological stability and incidence of side effects as recorded contemporaneously on bespoke audit proformas following each LISA procedure. We also assessed numbers needing endotracheal intubation within 24 hours post the LISA procedure.

**Results:**

120 neonates received midazolam premedication for LISA and had a completed proforma in the study period. In 119/120 (99.2%) cases, the procedure was deemed successful. Rates of side effects overall were: surfactant reflux n=22 (18.5%); bradycardia (heart rate <100/min) n=19 (16.0%); apnoea n=29 (24.4%); oxygen desaturation (SaO_2_<80%), n=56 (47.1%). Only 9/119 (7.6%) babies required endotracheal intubation and ventilation within 24 hours of their first LISA dose.

**Conclusions:**

In our experience, routine midazolam premedication for LISA was associated with relatively low rates of physiological instability and a high rate of procedural success. Midazolam is a worthy potential candidate for formal study in comparative trials with other LISA premedication drugs, and against non-pharmacological LISA administration.

**WHAT IS ALREADY KNOWN ON THIS TOPIC**

- Less invasive surfactant administration (LISA) is a routine procedure in the neonatal unit but the ideal premedication drug for the procedure is unknown.

**WHAT THIS STUDY ADDS**

- Midazolam in premedication dosage 50–100 µg/kg before less invasive surfactant appears associated with a relatively low rate of short-term side effects and a high rate of procedural success.

**HOW THIS STUDY MIGHT AFFECT RESEARCH, PRACTICE OR POLICY**

- Midazolam is a worthy potential candidate for formal study in comparative trials with other LISA premedication drugs, and against non-pharmacological LISA administration.

## Introduction

 Less invasive surfactant administration (LISA), also known as minimally invasive surfactant administration, to treat respiratory distress syndrome reduces mechanical ventilation and risk of bronchopulmonary dysplasia in preterm infants.^1^ Premedication can relieve procedural pain associated with laryngoscopy, thereby facilitating LISA, but may have side effects. There is a current debate and no conclusive recommendation regarding whether sedation premedication should be given routinely for all infants undergoing LISA or else be used only selectively, such as in overtly active or distressed infants.^2 3^ Yet the use of analgesia and/or sedation can increase the comfort of the neonate and so may improve procedural success rates. The optimal premedication drug for LISA is unknown. An ideal LISA drug or drug combination would allow analgesia and sedation with rapid onset and short duration, would not suppress spontaneous respirations and would have favourable short-term and long-term safety profiles.

Our tertiary-level neonatal intensive care unit (NICU) previously used fentanyl for LISA premedication. However, due to our suboptimal experiences of its efficacy as a single-agent LISA premedication, in July 2019 our centre switched to use of midazolam for LISA premedication instead and has used this routinely ever since. This study reports a prospective audit of the safety and efficacy of midazolam use for LISA premedication over a 5 years and 5-month long period.

## Methods

Between July 2019 and December 2024, we prospectively audited neonates who routinely received midazolam for LISA in our NICU. Our unit clinical guideline specifies that neonates undergoing LISA receive midazolam 50–100 µg/kg intravenously 2–5 min prior to the laryngoscopy and passage of the LISA catheter. Atropine 20 µg/kg was optional. Curosurf surfactant 200 mg/kg was administered via a dedicated LISA catheter (Surfcath or Vygon). Data regarding each LISA procedure were recorded contemporaneously by the operator immediately post procedure on a bespoke audit proforma (online supplemental file 1), which was then filed in the medical records. From analysis of these forms and, where necessary, the medical notes, we reviewed rates of procedural success, predefined as successful LISA catheterisation and surfactant administration, and physiological stability. We also reviewed the incidence of side effects recorded during the LISA procedures and numbers needing endotracheal intubation within the subsequent 24 hours.

### Patient and public involvement

Patients and the public were not involved in the design, delivery or interpretation of this study.

## Results

**Figure 1:**
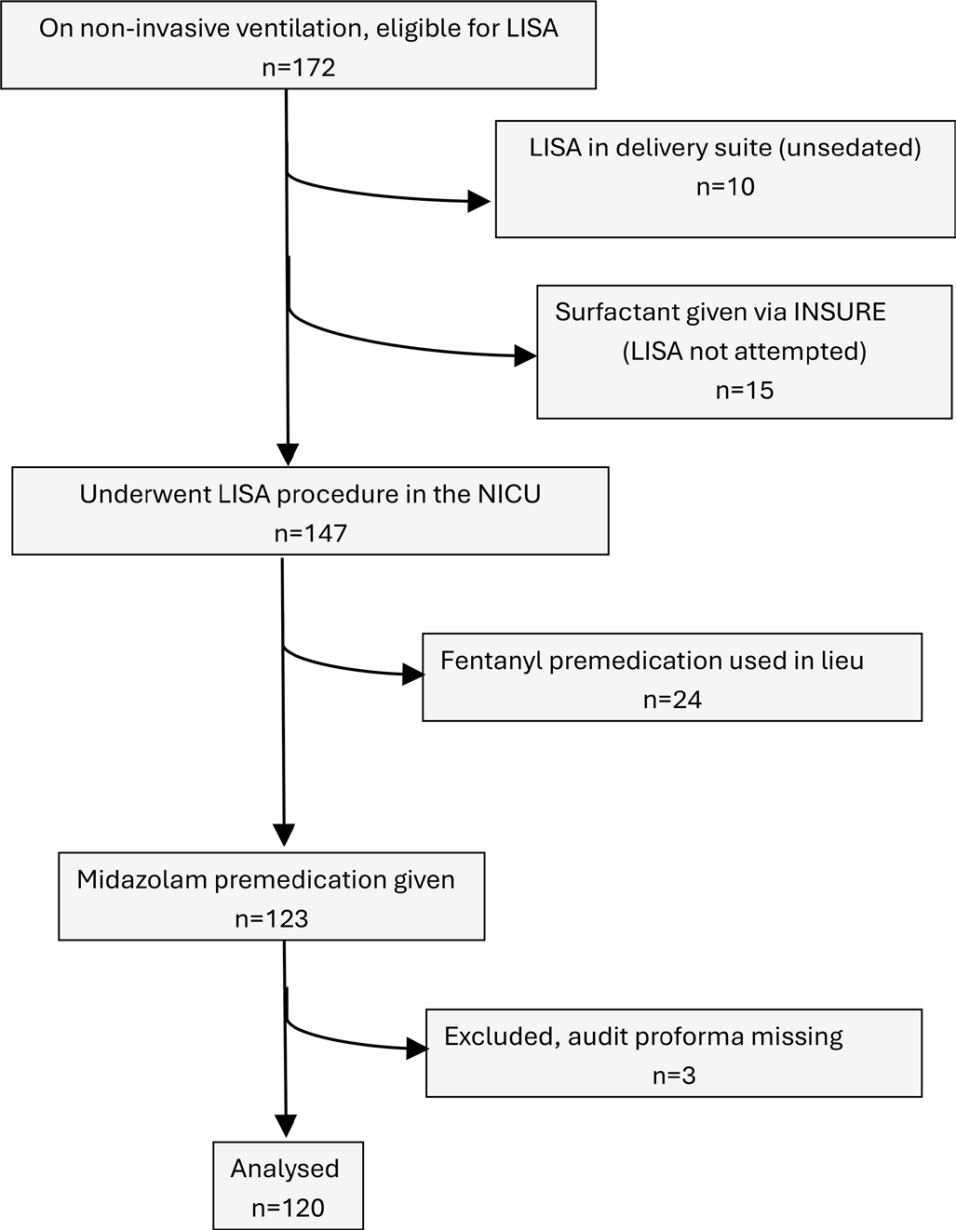
Study flow diagram. INSURE, Intubate-Surfactant-Extubate; LISA, less invasive surfactant administration; NICU, neonatal intensive care unit.

In the 5 years and 5-month long study period, there were 172 neonates potentially eligible for LISA (on non-invasive ventilation and assessed as requiring surfactant). Of these, 120 neonates received midazolam premedication for LISA within the NICU and had a completed proforma. Figure 1 shows the study flow diagram. Median gestational age (GA) was 32.0 weeks (IQR: 28.6–35.1; range: 24.2–38.4 weeks). N=58 received 50 µg/kg midazolam; n=58 received 100 µg/kg in total; n=1 received 50 µg/kg plus intravenous morphine 100 µg/kg; n=1 received 75 µg/kg midazolam in total; n=1 received 150 µg/kg midazolam in total; and n=1 received 300 µg/kg midazolam in total. Midazolam doses exceeding the 100 µg/kg limit specified in our guideline were given at the clinician’s discretion. Median postnatal age at LISA was 9 (IQR: 3–10) hours. Ventilatory support mode at the time of the LISA procedures was: nasal high flow, n=60; nasal continuous positive airway pressure, n=48; biphasic positive airway pressure, n=12. Seniority of operator performing the LISA procedure was: tier 1 (junior specialist NICU doctor/advanced neonatal nurse practitioner or clinical fellow), n=68; tier 2 (middle grade doctor/senior advanced neonatal nurse practitioner), n=51; tier 3 (consultant neonatologist), n=1. Median fraction of inspired oxygen (FiO_2_) immediately preceding LISA was 0.40 (IQR: 0.35–0.50) and 1-hour post-LISA was 0.30 (IQR: 0.23–0.40). In 119/120 (99.2%) cases, the procedure was deemed successful. Excluding four babies who were already in FiO2 0.21 at the time of LISA and who all remained so 1 hour post, the change in FiO2 from immediately before to 1 hour after successful LISA (n=115 babies) was median 0.10 (range: −0.29 to 0.70; IQR: 0.03 to 0.18). In 92/119 (77.3%) of LISA procedures deemed subjectively ‘successful’ by the operator, there was a decrease in FiO2 by ≥0.05 or to 0.21 (n=4 remained in FiO2 0.21 postprocedure). In the sole case considered unsuccessful, a 34.6-week gestation baby, a single dose of 75 µg/kg provided inadequate sedation and the LISA procedure was abandoned after two failed attempts. Only 9/119 (7.6%) babies required endotracheal intubation and ventilation within 24 hours of their first LISA dose.

Using our midazolam LISA protocol, overall 89 (74.2%) infants required only one attempt for successful LISA catheterisation; 24 (20.0%) required two attempts (n=1 abandoned), 6 (5.0%) required 3 attempts and 1 (0.8%) required 4 attempts. Of the 119 successful LISA events, 18 (15.1%) infants required one further dose of surfactant and only one (0.8%) of the infants required two further doses of surfactant within the subsequent 72 hours. Operator ratings scored on Likert scales (1–10, with 1=very difficult, 10=very easy) in response to the question on ease of LISA catheter insertion was median: 8 (IQR: 5 to 10) and in response to the question ‘How easy was it to perform the LISA procedure overall?’ was median: 7 (IQR: 5 to 10), with n=76 respondents in each case.

Rates of side effects contemporaneously recorded during the 119 successful LISA procedures were: surfactant reflux n=22 (18.5%); bradycardia (heart rate <100/min) n=19 (16.0%); apnoea n=29 (24.4%); oxygen desaturation (arterial blood oxygen saturation, SaO_2_<80%), n=56 (47.1%). Of the 29 infants with apnoea, 13 required stimulation only and 16 required stimulation and Neopuff mask intermittent positive pressure ventilation to resolve their apnoea. Table 1 presents the incidence of these side effects subgrouped according to GA. Surfactant reflux was highest in the term babies, while all other recorded side effects were highest in the extremely preterm subgroup. The incidence of recorded side effects was similar among the nine neonates that required intubation: surfactant reflux n=3 (33%), bradycardia n=3 (33%), apnoea n=5 (56%), desaturation <80% n=4 (44%). Regarding the sole neonate for whom midazolam sedation was unsuccessful for LISA, a single dose of 75 µg/kg failed to achieve sufficient sedation and the patient then received surfactant via the Intubate-Surfactant-Extubate method.

**Table 1:** Incidence of side effects associated with successful LISA after midazolam premedication with infants subgrouped according to birth gestational age

	Extreme preterm (≤27+6) N=14	Very preterm (28+0–31+6) N=46	Late preterm (32+0–36+6) N=43	Term (≥37+0) N=16
Surfactant reflux	2 (14%)	9 (20%)	6 (14%)	5 (31%)
Bradycardia	6 (43%)	8 (17%)	3 (7%)	2 (13%)
Apnoea	7 (50%)	17 (37%)	3 (7%)	2 (13%)
Desaturation	9 (64%)	22 (48%)	19 (44%)	6 (38%)

Data are n (%); bradycardia, heart rate <100/min; desaturation, arterial blood oxygen saturation decrease to <80%.

LISA, less invasive surfactant administration.

## Discussion

The use of premedication for LISA remains varied in neonatal units that have adopted the technique and is inconsistently reported in many studies on LISA.^1^ Our recent national UK survey showed 57% of all tertiary NICUs routinely give premedication(s) before LISA, though midazolam was used for LISA in only a single centre.^4^ In contrast, a survey of LISA practices among all tertiary NICUs in Austria, Germany and Switzerland in 2023 showed that midazolam was the preferred drug in 12 (20%) of the responding 60 units that used a sedative premedication for LISA.^5^ The midazolam dose used (50–100 μg/kg) was the same as that given to most babies in our own presently reported study.^5^ A national cohort study of premedication practices for LISA in 31 tertiary Polish NICUs included data from 16 patients that received midazolam, but no dose information was reported.^6^

A recent single centre audit retrospectively reviewed extremely preterm babies born at <28+0 GA who received LISA in the delivery room, mostly without any routine sedative premedication (given in <10% of cases).^7^ It reported a similar incidence of surfactant reflux (16% overall) but a lower incidence of apnoeas/bradycardias (19% overall), and oxygen desaturations (37% had SaO_2_<85%) compared with rates seen in our extremely preterm subgroup (table 1).^7^ Rates of side effects were likely underestimated due to the retrospective nature of that study.

Propofol has also been suggested as a suitable premedication for LISA, used in ~42% of Austrian/German/Swiss tertiary centres,^5^ though in only one UK tertiary NICU.^4^ Studying a cohort of very similar GA babies to our very preterm subgroup, one randomised control trial compared intravenous propofol 1 mg/kg as LISA premedication versus no premedication in 78 infants born at GA 28^+0^–32^+0^.^8^ This trial found a marginally higher incidence of bradycardia (21%) and a high incidence (91%) of oxygen desaturation (SaO_2_<85%) with propofol as compared with midazolam use in our very preterm subgroup (subgroup data in table 1).^8^

A limitation of our study is that we used a somewhat subjective measure of LISA success, namely as determined by the operator. While we additionally report pre and 1-hour post-LISA FiO2 values, others have used a more objective assessment of LISA success for primary outcome of successful LISA - including laryngoscopy-confirmed and recorded intratracheal LISA catheter position.^9^ Videolaryngoscopy was optional for LISA in our NICU during the audit period and we did not routinely record rates of videolaryngoscope use for the procedure.

The successful administration of pulmonary surfactant using the LISA technique while also maintaining the neonate’s comfort and physiological stability remains a significant clinical challenge.^1^ In our experience, routine midazolam premedication for LISA was associated with a high rate of procedural success. Our study adds to the limited literature for midazolam as a premedication for LISA. We conclude that midazolam is a worthy potential candidate for formal study in comparative trials with other LISA premedication drugs, and against non-pharmacological LISA administration. However, we recognise that midazolam’s excellent sedative and anxiolytic effects need to be weighed against its lack of any pure analgesic effect and its long half-life in preterm infants.^10^ We also acknowledge that early midazolam exposure has been cautioned in premature neonates due to recent dose-related concerns about impaired hippocampal growth and working memory outcomes.^11^ It is therefore imperative that future trials comparing premedication drugs study longer-term neurodevelopmental outcomes as well as short-term outcomes.

## Supplementary material

10.1136/bmjpo-2025-004219 online supplemental file 1

## Data Availability

Data are available on reasonable request.

## References

[1] Aldana-Aguirre JC, Pinto M, Featherstone RM (2017). Less invasive surfactant administration versus intubation for surfactant delivery in preterm infants with respiratory distress syndrome: a systematic review and meta-analysis. *Arch Dis Child Fetal Neonatal Ed*.

[2] Reynolds P, Bustani P, Darby C (2021). Less-Invasive Surfactant Administration for Neonatal Respiratory Distress Syndrome: A Consensus Guideline. Neonatology.

[3] Sweet DG, Carnielli VP, Greisen G (2023). European Consensus Guidelines on the Management of Respiratory Distress Syndrome: 2022 Update. Neonatology.

[4] Willgress L, Sava D, Daniels R (2025). Sedation and analgesia practices for less invasive surfactant administration, elective endotracheal intubation, and mechanical ventilation: a national UK survey. *Arch Dis Child Fetal Neonatal Ed*.

[5] Muehlbacher T, Boos V, Geiger LB (2024). Analgosedation for less-invasive surfactant administration: Variations in practice. Pediatr Pulmonol.

[6] Krajewski P, Szpecht D, Hożejowski R (2022). Premedication practices for less invasive surfactant administration — results from a nationwide cohort study. J Matern Fetal Neonatal Med.

[7] Klein R, Fastnacht L, Kribs A (2024). LISA Eligibility and LISA Success in Extremely Preterm Infants: A Single-Center Experience. Neonatology.

[8] Dekker J, Lopriore E, van Zanten HA (2019). Sedation during minimal invasive surfactant therapy: a randomised controlled trial. *Arch Dis Child Fetal Neonatal Ed*.

[9] Maiwald CA, Neuberger P, Franz AR (2021). Clinical evaluation of an application aid for less-invasive surfactant administration (LISA). *Arch Dis Child Fetal Neonatal Ed*.

[10] de Wildt SN, Kearns GL, Hop WC (2001). Pharmacokinetics and metabolism of intravenous midazolam in preterm infants. Clin Pharmacol Ther.

[11] Duerden EG, Guo T, Chau C (2023). Association of Neonatal Midazolam Exposure With Hippocampal Growth and Working Memory Performance in Children Born Preterm. Neurology.

