# Supplementary material for: Midazolam as premedication for less invasive surfactant administration: a prospective single-centre audit
Source: BMJ Paediatr Open. 2026 Apr 1;10(1):e004219. doi: 10.1136/bmjpo-2025-004219 (PMC13052648; doi:10.1136/bmjpo-2025-004219)
Supplement: online supplemental file 1 [file bmjpo-10-1-s001.docx]

**Lisa Audit Proforma**

**Please Complete Each Time Lisa Procedure Performed**

1. Date and Time of procedure: ……………………………… Time………………hrs
2. Baby’s Hospital Number: _____________________________
3. Name and grade of person performing LISA:_______________________________
4. Number of times you have personally done the LISA procedure before:
5. Dose of surfactant given (mg/kg):
6. Drugs used for this LISA procedure:

| **Drug** | **Given**  **Yes/No?** | **Dose used** | **Number of doses given** |
| --- | --- | --- | --- |
| Midazolam |  | mcg/kg |  |
| Morphine |  | mcg/kg |  |
| Fentanyl |  | mcg/kg |  |
| Atropine |  | mcg/kg |  |

1. Were the following drugs needed?

Suxamethonium yes no

Naloxone yes no

1. Ventilator mode at time of LISA *(tick)*:

nasal CPAP

BiPAP

Vapotherm

Other ……...*………………*

1. Was LISA successful? Yes No *if not, state why not*

*……………………………………………………………………………………………*

*……………………………………………………………………………………………*

*……………………………………………………………………………………………*

*……………………………………………………………………………………………*

1. How quickly did O_2_ requirement fall after LISA? Slowly Quickly
2. FiO_2_ before procedure: FiO_2_ ~1 hr after procedure:
3. Were there any associated adverse effects? *(tick)*:

|  | Yes | No |
| --- | --- | --- |
| Visible Oropharyngeal surfactant reflux |  |  |
| Bradycardia of <100/min |  |  |
| Apnoea |  |  |
| Desaturation (SaO_2_ <80%) |  |  |

1. Did baby require intubation following LISA? Yes No
2. How easy did you find the LISA catheter to use/insert? *(circle)*

**Very Very**

**difficult Neutral easy** |_______|_______|_______|_______|_______|_______|_______|_______|_______|

1. How easy was it to perform the LISA procedure overall? *(circle)*

**Very Very**

**difficult Neutral easy** |_______|_______|_______|_______|_______|_______|_______|_______|_______|

1. Please add below any other comments on your experience of LISA if you wish or suggest any improvements to our guideline.

*…………………………………………………………………………………………………………………………………………………………………………………*

*……………………………………………………………………………………………*

*………………………………………………………………………………………*

*…………………………………………………………………………………………*

*Thank you for taking the time to complete this audit questionnaire.*
