# Supplementary material for: Midazolam as premedication for less invasive surfactant administration: a prospective single-centre audit
Source: BMJ Paediatr Open. 2026 Apr 1;10(1):e004219. doi: 10.1136/bmjpo-2025-004219 (PMC13052648; doi:10.1136/bmjpo-2025-004219)
Supplement: Supplementary file 2 [file bmjpo-2025-004219.reviewer_comments.pdf]

PEER REVIEW HISTORY

BMJ Paediatrics Open publishes all reviews undertaken for accepted manuscripts. Reviewers are asked to complete a checklist review form and are provided with free text boxes to elaborate on their assessment. These free text comments are reproduced below.

ARTICLE DETAILS

**TITLE (PROVISIONAL)**	Midazolam as pre-medication for less invasive surfactant administration: a prospective single-centre audit
**AUTHORS**	Willgress, Liam<br>Sava, Dina<br>Selim, Fatma<br>Wells, Zina<br>Clarke, Paul

VERSION 1 – REVIEW

**REVIEWER NAME**	Maiwald, Christian Achim
**REVIEWER AFFILIATION**	University Hospital Tübingen, Department of Neonatology
**REVIEWER CONFLICT OF INTEREST**	Invented Neofact application aid (catheter) for LISA and therefore participates in their turnover. This will not influence the evaluation of this study.
**AI DISCLOSURE**	No, I have not used AI
**DATE REVIEW RETURNED**	12-Nov-2025

**GENERAL COMMENTS**	Thank you for selecting me as a reviewer for this very interesting manuscript. The topic is very well suited to the journal, certainly of interest to the readership and the manuscripts is also easy to understand. Nevertheless, I have a few comments to the authors:<br><br>Major Comments:<br>1. The primary outcome (based on the assessor's evaluation only) is a little weak. In other feasibility studies on LISA (e.g. 10.1136/archdischild-2020-319792), success was based on more measurable criteria (e.g. video laryngoscopic visualisation of the correct catheter position or FiO2 drop). The latter was recorded by the study form, so that at least both success rates (user assessment and FiO2-Drop) should be stated.<br><br>2. Similarly, the number of additional surfactant administrations during the first 72 hours gives a rough indication of whether there were any unsuccessful treatments (taking into account that there are, of course, infants who inactivate surfactant for other reasons and therefore require multiple administrations, but rates should be at least comparable).<br><br>3. Another criterion for how well a drug improves the LISA-procedure could also be the success rate of catheterisation in the first laryngoscopy attempt (if documented).<br><br>4. Additionally and according to the form in the appendix, users assessed how well the infant was sedated and how well the procedure was tolerated. However, these results are not reported, even though they would provide the reader with a more detailed assessment and potentially make the results more comparable with

	later studies.<br><br>5. The LISA-experience of the physicians performing the procedure and ventilator mode at time of LISA was also recorded but not reported. Both topics are of interest. The latter in particular could also give an impression of the extent to which midazolam-induced hypoventilation could be covered.<br><br>6. In the introduction, the authors describe what an optimal drug for LISA should be like, but they do not discuss the extent to which midazolam fails to meet these criteria. In my opinion, midazolam is not the most optimal drug for multiple reasons (e.g., it has no analgesic effect, is potentially neurotoxic (which is discussed) and its duration of action can be extremely long in premature infants (elimination half-life 6-12h; SmPC Dormicum)). The missing restrictions should also be discussed.<br><br><br>Minor Comments:<br>1. The recruitment periods at the end of the introduction and the beginning of the methods section are duplicated and are therefore unnecessary in one place.<br><br>2. Results: Maybe you could figure out the total number of LISA administrations (without midazolam and incompleted proforma) so that readers can conclude how high the inclusion rate in the study was. Possibly, a study flow diagram could also be helpful.<br><br>3. Discussion, first paragraph: More information (including common dosages used) can be found here: Muehlbacher, Pediatric Pulmonology, 2023 (https://doi.org/10.1002/ppul.26826) or Maiwald, Neonatology, 2025 (https://doi.org/10.1159/000539302).

**REVIEWER NAME**	Gowda, Harsha
**REVIEWER AFFILIATION**	University Hospitals Birmingham NHS Foundation Trust, Neonatal Medicine
**REVIEWER CONFLICT OF INTEREST**	No Conflict of Interest
**AI DISCLOSURE**	No, I have not used AI
**DATE REVIEW RETURNED**	29-Dec-2025

**GENERAL COMMENTS**	This is a well-structured prospective audit describing the safety and efficiency of midazolam during LISA. However, there are several issues that need to be addressed:<br><br>1, Abstract results (page 3, line 51):<br>The percentage is incorrect. It should read 9/119 (7.6%).<br><br>2, Results section (page 7, line 37):<br>Again, this should be corrected to 9/119.<br><br>3, Side effects and comparison group:<br>The reported side effects—apnoea (25%) and desaturations (46%)—are significant. Although the authors compare their findings with delivery-suite LISA data, the mean gestational age in those studies is <28 weeks, whereas the mean gestational age in this audit is 32 weeks. Infants <28 weeks are inherently more prone to apnoea due to immaturity. A comparison with cohorts of similar

	gestational age and/or with other premedications would be more appropriate and strengthen the conclusions.<br><br>4, Management of apnoea:<br>The manuscript does not describe how episodes of apnoea were managed. Please clarify whether interventions included stimulation, mask ventilation, or other respiratory support.<br><br>5, Use of sedation during LISA:<br>Current recommendations suggest using sedation selectively—only in active or distressed infants—rather than routinely for all infants undergoing LISA. This point should be acknowledged and discussed, particularly in the context of the reported side effects

VERSION 1 – AUTHOR RESPONSE

**BMJ Paediatrics Open - Decision on Manuscript ID bmjpo-2025-004219**	
Reviewer: 1<br>Dr. Christian Achim Maiwald, University Hospital Tübingen	
**Comments to the Author**<br>Thank you for selecting me as a reviewer for this very interesting manuscript. The topic is very well suited to the journal, certainly of interest to the readership and the manuscripts is also easy to understand. Nevertheless, I have a few comments to the authors:	We thank Dr Maiwald very much for reviewing our manuscript and for helpful suggestions and constructive criticisms. We respond to each of his points below.
Major Comments:<br>1. The primary outcome (based on the assessor's evaluation only) is a little weak. In other feasibility studies on LISA (e.g. 10.1136/archdischild-2020-319792), success was based on more measurable criteria (e.g. video laryngoscopic visualisation of the correct catheter position or FiO2 drop). The latter was recorded by the study form, so that at least both success rates (user assessment and FiO2-Drop) should be stated.	We accept this criticism, that our mode of assessment was subjective and could have been more objective as in the way Dr Maiwald has suggested. We are grateful for these valuable points and suggestions.<br><br>We have now added as a limitation into our revision that we did not use a more objective outcome measure of ‘success’.<br><br>We have now also added the same objective success measure of decrease in FiO_2 by ≥0.05 or to 0.21 within 1 hour of LISA, this was the case for 92/119 babies. We have added the following sentences to the revised Results:<br><br>*“Excluding four babies who were already in FiO2 0.21 at the time of LISA and who all remained so 1 hour post, the change in FiO2 from immediately before to 1 hour after successful LISA (n=115 babies) was median 0.10 (range: -0.29 to 0.70; IQR: 0.03 to 0.18). In 92/119 (77.3%) of LISA procedures deemed subjectively ‘successful’ by the operator, there was a decrease in FiO2 by ≥0.05 or to 0.21 (n=4 remained in FiO2 0.21 post procedure).”*<br><br>We have added the following to the Discussion<br><br>*“A limitation of our study is that we used a somewhat subjective measure of LISA success, namely as determined by the operator. While we additionally report pre- and 1-hour post-LISA FiO2 values, others have used a more objective assessment of LISA success for primary outcome of successful LISA - including laryngoscopy-confirmed and*

	recorded intratracheal LISA catheter position.6 Videolarnyngoscopy was optional for LISA in our NICU during the audit period and we did not routinely record rates of videolaryngoscope use for the procedure."
2. Similarly, the number of additional surfactant administrations during the first 72 hours gives a rough indication of whether there were any unsuccessful treatments (taking into account that there are, of course, infants who inactivate surfactant for other reasons and therefore require multiple administrations, but rates should be at least comparable).	Thank you. We have reviewed the medical notes of all babies in the study to find this information, and now have the requested data available to include:<br><br>We have thus added the following sentence to the revised Results:<br><br>*"Of the 119 successful LISA events, 18 (15.1%) infants required one further dose of surfactant and only one (0.8%) of the infants required two further doses of surfactant within the subsequent 72 hours*."
3. Another criterion for how well a drug improves the LISA-procedure could also be the success rate of catheterisation in the first laryngoscopy attempt (if documented).	These data were recorded, thank you for encouraging us to include this. We have now added the following to out revised manuscript:<br><br>*"Using our midazolam LISA protocol, overall 89 (74.2%) infants required only one attempt for successful LISA catheterisation; 24 (20.0%) required two attempts (n=1 abandoned), 6 (5.0%) required 3 attempts, and 1 (0.8%) required 4 attempts. Of the 119 successful LISA events, 18 (15.1%) infants required one further dose of surfactant and only one (0.8%) of the infants required two further doses of surfactant within the subsequent 72 hours."*
4. Additionally and according to the form in the appendix, users assessed how well the infant was sedated and how well the procedure was tolerated. However, these results are not reported, even though they would provide the reader with a more detailed assessment and potentially make the results more comparable with later studies.	On review of our master datasheet and original source completed audit proformas, we find that the audit form used during the midazolam audit period had different questions for number (14.) and (15.) We apologise for this error in sending up a different version of the audit form that was revised subsequent to the audit period. We have now, for this revised submission replaced the previously submitted audit proforma version, and have now submitted the correct version that was actually used for the whole audit period. This version had the following questions for #14 and #15 with accompanying Likert scales 1-9 for use ratings:<br><br>14. How easy did you find the LISAcath® to use/insert? *(circle)*<br><br>15. How easy was it to perform the LISA procedure overall? *(circle)*<br><br>We have now included the summary statistics (median and range) for the responses received for these items. A new paragraph has been added into the revised manuscript, as follows:<br><br>" Operator ratings scored on Likert scales (1-10, with 1=very difficult, 10=very easy) in response to the question on ease of LISA catheter insertion was median: 8 (IQR: 5 to 10) and in response to the question 'How easy was it to perform the

	LISA procedure overall?' was median: 7 (IQR: 5 to 10), with<br><br>n=76 respondents in each case. ”<br><br><br>Although in this audit period we did not in fact ask specifically about operator perceived degree of successful sedation with the drug or how well the baby tolerated the LISA procedure with the premedication, we consider that some inference as to these items can be made from the number of repeat doses given and the latter now-reported item ‘how easy was it to perform the LISA procedure overall’, while accepting that the latter would vary according to an individual operator’s experience and skill and familiarity with the procedure, and - to a degree - seniority
5. The LISA-experience of the physicians performing the procedure and ventilator mode at time of LISA was also recorded but not reported. Both topics are of interest. The latter in particular could also give an impression of the extent to which midazolam-induced hypoventilation could be covered.	Thank you for these very useful comments.<br><br>In the revised Results, we have added details of the grade/seniority of operator performing the LISA procedure according to whether they were tier 1 (junior specialist NICU doctor/advanced neonatal nurse practitioner or clinical fellow), tier 2 (middle grade doctor/senior advanced neonatal nurse practitioner), or tier 3 (consultant neonatologist).<br><br>We have reviewed our data collection sheet and are have now added in the following into the revised manuscript results:<br><br>“Ventilatory support modes at the time of the LISA procedures were: nasal high flow, n=60; nasal continuous positive airway pressure, n=48; biphasic positive airway pressure, n=12.”
6. In the introduction, the authors describe what an optimal drug for LISA should be like, but they do not discuss the extent to which midazolam fails to meet these criteria. In my opinion, midazolam is not the most optimal drug for multiple reasons (e.g., it has no analgesic effect, is potentially neurotoxic (which is discussed) and its duration of action can be extremely long in premature infants (elimination half-life 6-12h; SmPC Dormicum)). The missing restrictions should also be discussed.	Thank you for these valid comments.<br>We have added the following into the revised Discussion, along with a supporting new citation for the long half life in preterm babies:<br><br>However, we recognise that midazolam’s excellent sedative and anxiolytic effects need to be weighed against its lack of any pure analgesic effect and its long half life in preterm infants.[7]
Minor Comments:<br>1. The recruitment periods at the end of the introduction and the beginning of the methods section are duplicated and are therefore unnecessary in one place.	Thank you for pointing out this redundancy. We have now deleted the first of the iterations from the Introduction.
2. Results: Maybe you could figure out the total number of LISA administrations (without midazolam and incompleted proforma) so that readers can conclude how high the inclusion rate in the study was. Possibly, a study flow diagram could also be helpful.	Thank you for this valuable suggestion. We have reviewed the requisite data and have now added a study flow diagram as a new Figure 1.
3. Discussion, first paragraph: More information (including common dosages used) can be found here: Muehlbacher, Pediatric Pulmonology, 2023 (https://doi.org/10.1002/ppul.26826) or	Many thanks to the pointers to these reports. We gratefully include both. In the first para of our revised discussion we have cited the Muehlbaher survey and midazolam dose used in the surveyed 20% of respondent NICUs that were using it for LISA premedication.

Maiwald, Neonatology, 2025 (https://doi.org/10.1159/000539302).	
**Reviewer: 2**<br>Dr. Harsha Gowda, University Hospitals Birmingham NHS Foundation Trust	
**Comments to the Author**<br>This is a well-structured prospective audit describing the safety and efficiency of midazolam during LISA. However, there are several issues that need to be addressed:	We thank Dr Gowda for reviewing our manuscript and for helpful comments.
1, Abstract results (page 3, line 51):<br>The percentage is incorrect. It should read 9/119 (7.6%).	We are grateful to Dr Gowda for spotting this significant error. It is now corrected in the revised Abstract.
2, Results section (page 7, line 37):<br>Again, this should be corrected to 9/119.	We are grateful to Dr Gowda for spotting this significant error. It is now corrected in the revised Results.
3, Side effects and comparison group:<br>The reported side effects—apnoea (25%) and desaturations (46%)—are significant. Although the authors compare their findings with delivery-suite LISA data, the mean gestational age in those studies is <28 weeks, whereas the mean gestational age in this audit is 32 weeks. Infants <28 weeks are inherently more prone to apnoea due to immaturity. A comparison with cohorts of similar gestational age and/or with other premedications would be more appropriate and strengthen the conclusions.	Thank you for the comment. However we have not made any significant revisions in response because we had **already** compared the rate of side effects in the cited delivery room LISA study (Klein et al.) with our extreme preterm subgroup (i.e. all <28 weeks' gestation, see Table 1).<br><br>As we had already written in our first submission:<br><br>"[Klein et al.] reported a similar incidence of surfactant reflux (16% overall) but a lower incidence of apnoeas/bradycardias (19% overall), and oxygen desaturations (37% had SaO_2 <85%) compared with rates seen in our extremely preterm sub-group (**Table 1**)."<br><br>To make this a bit clearer to the reader, we have amended slightly to say "(subgroup data in **Table 1**)".
4, Management of apnoea:<br>The manuscript does not describe how episodes of apnoea were managed. Please clarify whether interventions included stimulation, mask ventilation, or other respiratory support.	Thank you for this point. On reviewing our raw datasheet we noticed an error: there were actually 29 and not 30 babies who had apnoea complicating their successful LISA. We apologise for the inaccuracy and have corrected the figures in the Abstract and Results accordingly.<br><br>In answer to the referee's query: we have reviewed our source data and have included the following addition into our revision:<br><br>*"Of the 29 infants with apnoea, 13 required stimulation only and 16 required stimulation and Neopuff mask intermittent positive pressure ventilation to resolve their apnoea."*
5, Use of sedation during LISA:<br>Current recommendations suggest using sedation selectively—only in active or distressed infants—rather than routinely for all infants undergoing LISA. This point should be acknowledged and discussed, particularly in the context of the reported side effects	We are not aware of any unanimous or major current recommendation to use sedation *only selectively*. Indeed we would find such a selective recommendation to be odd because attempting the procedure in a non sedated infant will inevitably lead to some babies becoming distressed, and by then it is arguably too late for them to use sedation.<br><br>We have reviewed current UK/European opinion on the issue and added two new citations to the Introduction of our revised manuscript to indicate the current lack of consensus regarding the issue of routinely sedating for LISA - neither of the two newly cited papers state a preference or current recommendation for using sedation only selectively:

	i) Consensus statement of 9 UK neonatologists on practice of LISA where they state:<br><br>*"Sedation should particularly be considered in babies >32 weeks, but the need for sedation should be considered in all babies." (Ref. Reynolds P, et al. Less-Invasive Surfactant Administration for Neonatal Respiratory Distress Syndrome: A Consensus Guideline. Neonatology. 2021;118(5):586-592. doi: 10.1159/000518396.)*<br>*Downloadable from here: https://www.researchgate.net/publication/354583569_Less-Invasive_Surfactant_Administration_for_Neonatal_Respiratory_Distress_Syndrome_A_Consensus_Guideline/link/62e52e7b3c0ea878876aa0d0/download)*<br><br>*ii) Sweet DG, et al. European Consensus Guidelines on the Management of Respiratory Distress Syndrome: 2022 Update. Neonatology. 2023;120(1):3-23. doi: 10.1159/000528914. https://karger.com/neo/article-pdf/120/1/3/3965644/000528914.pdf*<br><br>Furthermore, we would note that the British Association for Perinatal Medicine does not have an official position on whether to use sedation routinely or only selectively. *(see The British Association of Perinatal Medicine Neonatal Airway Safety Standard A Framework for Practice April 2024, BAPM_Airway_Standards_Full_July24.pdf*<br><br>*and the BAPM LISA checklist: https://hubble-live-assets.s3.eu-west-1.amazonaws.com/bapm/file_asset/file/2547/Appendix_F_-_LISA_checklist.pdf)*

VERSION 2 – REVIEW

**REVIEWER NAME**	Maiwald, Christian Achim
**REVIEWER AFFILIATION**	University Hospital Tübingen, Department of Neonatology
**REVIEWER CONFLICT OF INTEREST**	Invented Neofact application aid (catheter) for LISA and therefore participates in their turnover. This will not influence the evaluation of this study.
**AI DISCLOSURE**	No, I have not used AI
**DATE REVIEW RETURNED**	07-Feb-2026

**GENERAL COMMENTS**	Thank you very much for implementing the points mentioned and for the acknowledgement at the end of the manuscript. I would only ask you to review the citations again, as the numbering does not match the list below. Otherwise, I have no further objections. Congratulations on your successful work.

**REVIEWER NAME**	Gowda, Harsha
**REVIEWER AFFILIATION**	University Hospitals Birmingham NHS Foundation Trust, Neonatal Medicine
**REVIEWER CONFLICT OF INTEREST**	No Conflict of Interest
**AI DISCLOSURE**	No, I have not used AI

**DATE REVIEW RETURNED**	19-Feb-2026

**GENERAL COMMENTS**	Authors have addressed all the comments.
